# Influence of an educational program utilizing VAK and Kolb’s learning theories on basic cardiopulmonary resuscitation knowledge and practices among private home nurses in Qatar

**DOI:** 10.1016/j.resplu.2025.101071

**Published:** 2025-08-19

**Authors:** Mohamed Elsayed Saad Aboudonya, Hoda Diab Fahmy Ibrahim, Safaa R. Osman

**Affiliations:** a Hamad International Training Center, Qatar; b Faculty of Nursing, Assiut University, Egypt

**Keywords:** Attitude, Cardiopulmonary resuscitation, Educational Program, Knowledge, Kolb’s experiential learning model, VAK learning, Private home-care nursing

## Abstract

**Background:**

Basic CPR is vital for home nurses, yet knowledge and practice gaps remain. Theory-based training can enhance skill effectiveness.

**Aim:**

This study aimed to evaluate the influence of VAK and Kolb’s learning theories on basic cardiopulmonary resuscitation knowledge and practices among private home nurses in Qatar.

**Methods:**

Quasi–experimental pre–/post study. One–hundred–thirty–four nurses were randomized to VAK and Kolb group (each n = 67). A learning–style inventory, CPR knowledge questionnaire, basic life and automated external defibrillator checklists were completed at baseline, immediately post–training, and at 6 and 9  months. The intervention composed of multimodal training program combined a 1–h multimedia lecture incorporating case-based scenarios with a 3–h European Resuscitation Council four–stage workshop customized to cover all learning styles in both groups.

**Results:**

Participants were predominantly female (88.1 %), aged 35–44  years (44.8 %) and bachelor–prepared (59.7 %). Immediately after training, satisfactory CPR knowledge rose from 35 % to 90 %, BLS competence from 1.5 % to 100 % and AED operation from 23 % to 100 % (all p < 0.001). Retention fell sharply at 6  months (25.6 %, 25.6 % and 60.5 %, respectively) and only partly recovered by 9  months (53.3 %, 27.4 % and 71.4 %). Visual, auditory and concrete–experience learners showed the steepest decline, whereas kinesthetic and reflective–observer learners maintained the highest performance.

**Conclusion:**

Retention patterns differed sharply across learning styles. Visual, auditory, and concrete–experience nurses reached near–perfect scores right after training but lost much of those gains within six months. By nine months, kinesthetic (VAK) and reflective–observer (Kolb) learners still led CPR and AED performance, while visual, active–experimenter, and abstract–conceptualizer groups showed the steepest drop–offs. Sustained competence therefore hinges on both refresher timing and the cognitive–sensory mode through which skills were first acquired.

**Recommendations:**

Use VAK and Kolb profiling during initial competency checks to tailor refresher frequency (quarterly low-dose sessions for visual, auditory, and concrete learners; semi-annual for kinesthetic and reflective learners), conduct an annual full-skills audit, and assign a dedicated Resuscitation Officer to coordinate and monitor these activities.

## Graphical abstract

**Figure:**
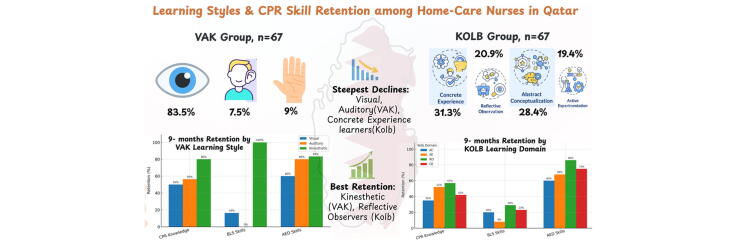

## Introduction

Out-of-hospital cardiac arrest (OHCA) is a major global health concern, accounting for 15–20 % of all deaths worldwide.^1^, ^2^ It is a life-threatening emergency that requires immediate intervention, with early CPR and defibrillation being critical for survival. CPR maintains oxygen-rich blood flow to vital organs until professional help can restore normal heart function; without prompt action, irreversible brain damage can occur within minutes.^1^, ^2^ Globally, OHCA occurs at an average rate of fifty-five cases per 100,000 person-years.^3^ In Qatar, a national study reported an annual crude incidence of 23.5 per 100,000 EMS-attended cardiac arrests and a standardized rate of 87.8 per 100,000, with Qataris comprising 20 % of cases — highlighting the need for better pre-hospital care and public CPR training.^4^ Data from Gulf Cooperation Council countries show that most OHCAs (54–85 %) happen at home, underscoring the importance of training likely first responders, such as private home nurses.^5^, ^6^

The private sector employs about 5846 nurses, roughly 800 of whom give in-home care and are often first on the scene during OHCA. Unlike public institutions such as Hamad Medical Corporation, the private sector lacks dedicated CPR trainers, creating gaps in preparedness.^7^, ^8^, ^9^ Although healthcare providers in Qatar must keep CPR certificates current for licensure, evidence shows that skills decline within two weeks and knowledge within 1–6 months after training.^10^ Educational frameworks like Kolb’s Experiential Learning Theory and the VAK (Visual, Auditory, Kinesthetic) model enhance knowledge and hands-on ability in healthcare training. Kolb through cyclical experience, reflection, conceptualization, and experimentation^11^; VAK by tailoring training to sensory preferences, promoting retention and application.^12^

This study tackles a critical deficit in Qatar’s system by focusing on the CPR readiness of private home nurses—often the first responders to OHCA.^3^, ^4^ By comparing VAK and Kolb-based training, it seeks to boost their emergency competence and improve community survival outcomes.^11^, ^13^ Furthermore, this work advances learner–centered CPR education research. Although the VAK and Kolb frameworks are well established in healthcare, their specific use for CPR training among Qatar’s private home–care nurses remains under–investigated and could illuminate strategies to enhance retention, knowledge transfer, and practical proficiency.^14^

### Significance of the study

CPR training enables Qatar’s private home-care nurses to recognize cardiac arrest swiftly, deliver effective resuscitation, and meet certification regulatory requirements that advance their careers, while also educating families and boosting community preparedness.^15^ This competence is critical given the high-acuity, round-the-clock caseload—ventilator-dependent, palliative, and other complex patients—managed by private services, where arrest risk is heightened.^16^

### Aim of the study

This study aimed to evaluate the impact of VAK and Kolb’s learning theories on basic cardiopulmonary resuscitation (CPR) knowledge and practices among private home-care nurses in Qatar.

It was hypothesized that nurses’ preferred learning styles—classified by the VAK sensory modality model or Kolb’s experiential learning cycle—would be significantly associated with their CPR knowledge and performance. Alternatively, the null hypothesis proposed no statistically significant association between learning-style classifications and CPR outcomes.

### Research design and subjects

The study employed a quasi-experimental design with pre-test and post-test. This study was conducted on the private home care nurses in Qatar who are coordinated by a private nursing service branch in Hamad Medical Corporation, with a total number of eight hundred private home care nurses.

### Sampling

**Fig. 1:**
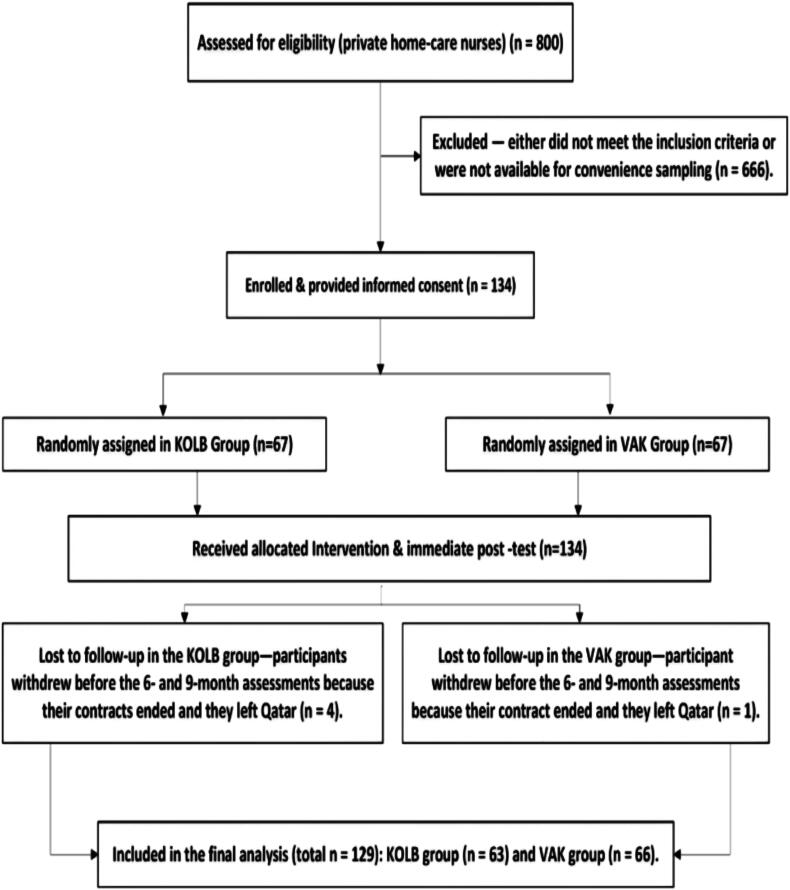
Flowchart of the sampling methodology and study procedures for the pretest and post–test assessments (immediate, 6–month, and 9–month).

G*Power (d = 0.30, α = 0.05, power = 0.95) indicated a required sample of 134 from ∼800 eligible nurses. Simple random sampling selected 134 private home-care nurses **[**see Fig. 1**],** The allocation sequence was generated and implemented by the principal investigator**,** who were then evenly allocated to VAK or Kolb CPR-training groups (67 each). Six and nine-month follow-ups saw five contract-related dropouts (1 VAK, 4 Kolb), leaving 129 participants for analysis (VAK = 66, Kolb = 63). Eligible participants are volunteer private home-care nurses in Qatar who had no formal CPR training within the past year.

### Tools of the study

**Tool I – Knowledge Questionnaire included *Demographics*** – age, sex, education, experience, and Kolb LSI – 80-item licensed survey (CE, RO, AC, AE) classifying sixty-seven nurses into Converger, Diverger, Assimilator, or Accommodator styles. VAK questionnaire – 30-item licensed instrument completed by another sixty-seven nurses, categorizing visual, auditory, kinesthetic, or multimodal learners. Finally, CPR Knowledge questionnaire– 15 multiple-choice items on basic-life-support concepts; ≥60 % = satisfactory**.**

**Tool II – CPR Skills Checklist:** Nine ERC-based actions subdivided into thirty steps; performances video-recorded pre/post-training. Each correct step = 1 point; ≥60 % signals adequate skill.

**Tool III – AED Skills Checklist**: Ten ERC-based tasks (arrest confirmation, pads placement, shock delivery, safety) scored 0/1; ≥60 % denotes satisfactory. Sessions were likewise video recorded.

**Validity & Reliability**: Researcher-developed sections (Tool I-Part 4, Tools II–III) were content-validated by five Hamad International Training Center faculty. Overall internal consistency was good (Cronbach’s α = 0.88). Licensed instruments (Kolb LSI, VAK) were used with formal permission.

**Pilot Study:** A pilot study involving fourteen private home-care nurses (10 %) was conducted to evaluate the clarity and flow of the tools and to estimate data collection time. Minor revisions were made based on feedback. Pilot data were excluded from the final analysis.

**Preparation Phase:** All required approvals were obtained from Assiut University Hospital, Qatar’s Private Nursing Sector, and Hamad Medical Corporation. Ethical clearance was granted. After informed consent, 134 nurses were randomly assigned to Kolb and VAK groups (n = 67 each), with learning styles assessed via LSI and VAK inventories. Experts reviewed the developed educational program. Session schedules, training venue, audiovisual aids, and handouts were also arranged during this phase.

**Implementation:** Started on September 2024, purposefully employed a multimodal design to cover all VAK and Kolb learning styles. Nurses were split into subgroups^22^, ^23^ participants each] and completed three sequential sessions. First, over two hours, the investigator introduced the study, gained written consent, and administered individual pre-tests to establish baseline CPR competence. Next, a one-hour PowerPoint lecture, interwoven with brief video clip and reflective scenario cases, reinforced the ERC chain of survival and the value of early CPR and prompt defibrillation. Finally, a three-hour practical station adopted the ERC four-stage skills-teaching method—real time demonstration, narrated demonstration with discussion, instructor-guided walk-through, and participant-led performance with feedback. European Resuscitation Council-certified ALS instructors assisted throughout data collection.

The multimodal program was deliberately structured to engage every major learning style in both groups **[**See Supplementary Table S1**]**: an interactive PowerPoint with embedded full-scenario video addressed visual and auditory modalities while supporting Kolb’s Abstract-Conceptualization and Reflective-Observation stages; facilitated discussions of real-life reflective cases deepened auditory/visual processing and promoted further reflection and concept formation; finally, the ERC four-stage skills demonstration (real-time demo, narrated demo, guided practice, independent performance with feedback) provided the kinesthetic, hands-on Concrete-Experience and Active-Experimentation phases. Taken together, these three elements span the full Kolb learning cycle and deliver content through all VAK channels, ensuring that nurses—regardless of individual preference—receive information multiple ways and have the opportunity to practice, reflect, and internalize essential CPR competencies.

**Evaluation Phase:** Outcomes were assessed using the same tools immediately post-intervention and at 6 and 9 months to measure the program's impact over time.

**Ethical approvals:** Ethical approval was initially obtained from the Faculty of Nursing Ethical Research Committee, Assiut University, Egypt (**Council Meeting 163, 18 February 2024**), followed by approval from the Medical Research Center at Hamad Medical Corporation, Qatar (**Protocol MRC-01-24-266**). The study was prospectively registered on ClinicalTrials.gov **(NCT06865690**; https://www.clinicaltrials.gov/study/NCT06865690?locStr=Qatar&country=Qatar&rank=9).

### Statistical analysis

Participants were randomly assigned to either VAK- or Kolb-based groups prior to receiving multimodal CPR training. Baseline characteristics were summarized using means ± standard deviation or medians with interquartile ranges, based on distribution assessed via Shapiro–Wilk tests and Q–Q plots. Categorical variables were reported as frequencies and percentages, with appropriate between-group comparisons using t-tests, Mann–Whitney U, χ2, or Fisher’s exact tests. Longitudinal outcomes for CPR knowledge (0–15), BLS skills (0–30), and AED skills (0–10) were analyzed using linear mixed-effects models, specifying fixed effects for time (categorical; pre-test as reference) and a random intercept for each participant. Models were adjusted for age, sex, education, real-life CPR exposure, and years of clinical experience. Results are presented as unstandardized β coefficients with 95 % confidence intervals and two-sided p-values (significance set at p < 0.05). Model assumptions were evaluated graphically, and multicollinearity was assessed using variance inflation factors. Missing data were addressed under the mixed-model missing-at-random assumption. All analyses were conducted using IBM SPSS Statistics (Version 30.0, 2025).

## Results

Notable differences in learning-style distribution are evident (Table 1**)**: Kolb participants are spread across four styles, whereas the VAK group is predominantly visual (83.5 %). Despite this programmed divergence, baseline characteristics such as gender, age, work setting, prior CPR exposure, and certifications remain largely similar (p ≥ 0.215). However, the VAK group has a higher proportion of diploma-level nurses (49.3 % vs 31.3 %, p = 0.035) and, with 52.2 % in the 6–10-year experience range, fewer in the 1–5-year band (p = 0.063).

**Table 1:** Baseline demographic characteristics of Kolb vs VAK groups.

**Variable**	**Category**	**Kolb (n = 67)**	**VAK (n = 67)**	***P*-value**
**Preferred learning style**	Visual	0 (0.0 %)	56 (83.5 %)	<0.001**
	Auditory	0 (0.0 %)	5 (7.5 %)	
	Kinesthetic	0 (0.0 %)	6 (9.0 %)	
	Abstract Conceptualization	19 (28.4 %)	0 (0.0 %)	
	Active Experimentation	13 (19.4 %)	0 (0.0 %)	
	Reflective Observation	14 (20.9 %)	0 (0.0 %)	
	Concrete Experience	21 (31.3 %)	0 (0.0 %)	

**Gender**	Male	7 (10.4 %)	9 (13.4 %)	0.791
	Female	60 (89.6 %)	58 (86.6 %)	

**Age (years)**	25–34	29 (43.3 %)	38 (56.7 %)	0.166
	35–44	35 (52.2 %)	25 (37.3 %)	
	45–54	3 (4.5 %)	2 (3.0 %)	
	≥55	0 (0.0 %)	2 (3.0 %)	

**Education**	Diploma	21 (31.3 %)	33 (49.3 %)	0.035*
	Bachelor	46 (68.7 %)	34 (50.7 %)	

**Experience (years)**	<1	1 (1.5 %)	0 (0.0 %)	0.063
	1–5	27 (40.3 %)	17 (25.4 %)	
	6–10	19 (28.4 %)	35 (52.2 %)	
	11–15	16 (23.9 %)	11 (16.4 %)	
	>15	4 (6.0 %)	4 (6.0 %)	

**CPR training <1 yr**	Yes	56 (83.6 %)	59 (88.1 %)	0.458
	No	11 (16.4 %)	8 (11.9 %)	

**BLS certificate**	Yes	23 (34.3 %)	25 (37.3 %)	0.719
	No	44 (65.7 %)	42 (62.7 %)	

**ILS certificate**	Yes	56 (83.6 %)	53 (79.1 %)	0.506
	No	11 (16.4 %)	14 (20.9 %)	

**ALS certificate**	Yes	28 (41.8 %)	25 (37.3 %)	0.596
	No	39 (58.2 %)	42 (62.7 %)	

**Real-life arrest experience**	Yes	43 (64.2 %)	42 (62.7 %)	0.858
	No	24 (35.8 %)	25 (37.3 %)	

* = significant (*P* < 0.05);

** = highly significant (*P* < 0.01).

All subgroups demonstrated a significant time-effect (Table 2**;** Cochran’s Q, df = 3; all p < 0.01). Immediately post-training ≥ 80 % passed. By 6 months, kinesthetic learners retained best (50 %; p = 0.002), visual collapsed to 3.6 % then rebounded to 56.4 % at 9 months (p < 0.001), and auditory stayed 40–50 % (p = 0.001). VAK overall fell from 92.5 % to 10.6 % at 6 months and rose to 57.6 % at 9 months (p < 0.001). In Kolb, reflective learners declined least (78.6 % to 35.7 % and 57.1 %), concrete the most (13.6 % at 6 months; p < 0.001). Overall, Kolb outperformed VAK at 6 months (23.8 % vs 10.6 %) but not at 9 months (46.0 % vs 57.6 %).

**Table 2:** Total level of satisfactory CPR knowledge retention by learning-style framework.

**Framework**	**Learning Style**	**Pre–Test**	**Immediate Post**	**6–Mo**	**9–Mo**	***p*–value**
		**VAK n = 67**, **Kolb n = 67**	**VAK n = 67**, **Kolb n = 67**	**VAK n = 66,** **Kolb n = 63**	**VAK n = 66,** **Kolb n = 63**	
**VAK**	Visual	22 (39.3 %)	52 (92.9 %)	2 (3.6 %)	31 (56.4 %)	<0.001*
	Auditory	1 (20.0 %)	4 (80.0 %)	2 (40.0 %)	3 (50.0 %)	0.001*
	Kinesthetic	1 (16.7 %)	6 (100 %)	3 (50.0 %)	4 (80.0 %)	0.002*
	**VAK Total**	**24 (35.8 %)**	**62 (92.5 %)**	**7 (10.6 %)**	**38 (57.6 %)**	<0.001*

**Kolb**	Abstract Conceptualization (AC)	9 (47.4 %)	16 (84.2 %)	4 (21.1 %)	9 (52.9 %)	<0.001*
	Active Experimentation (AE)	4 (30.8 %)	11 (84.6 %)	3 (25.0 %)	5 (41.7 %)	<0.001*
	Reflective Observation (RO)	8 (57.1 %)	11 (78.6 %)	5 (35.7 %)	8 (57.1 %)	<0.001*
	Concrete Experience (CE)	14 (66.7 %)	20 (95.2 %)	3 (13.6 %)	7 (35.0 %)	<0.001*
	**Kolb Total**	**35 (52.2 %)**	**58 (86.6 %)**	**15 (23.8 %)**	**29 (46.0 %)**	<0.001*

* Highly significant (p < 0.01).

A significant time-effect was again observed across all subgroups (Table 3**;** Cochran’s Q, all p ≤ 0.002). Immediately post-training every cohort achieved 100 % satisfactory BLS. By 6 months, kinesthetic learners still scored 100 % (p = 0.002), visual dropped to 21.8 % and auditory to 0 % (p = 0.001); the VAK total fell to 27.3 % (p < 0.001). Within Kolb, Reflective Observation retained the highest performance (35.7 %), Concrete Experience the lowest (15.8 %), yielding a 23.8 % framework total (p < 0.001). At 9 months kinesthetic remained perfect, visual edged down to 16.4 %, auditory stayed at 0 %, and VAK settled at 21.2 %. Kolb dipped to 19 %, led again by Reflective Observation (28.6 %) and trailed by Active Experimentation (8.3 %).

**Table 3:** Total level of Satisfactory BLS Performance by Learning Style.

**Model**	**Learning Style**	**Pre Test**	**Immediate Post Test**	**6 Mo Post Test**	**9 Mo Post Test**	***p*–value**
		**VAK n = 67,Kolb n = 67**	**VAK n = 67,Kolb n = 67**	**VAK n = 66,Kolb n = 63**	**VAK n = 66,Kolb n = 63**	
**VAK**	Visual	3.6 %	100 %	21.8 %	16.4 %	<0.001*
	Auditory	0 %	100 %	0 %	0 %	0.001*
	Kinesthetic	0 %	100 %	100 %	100 %	0.002*
	**Total**	3.0 %	100 %	27.3 %	21.2 %	<0.001*

**Kolb**	Abstract Conceptualization (AC)	0 %	100 %	22.2 %	23.5 %	<0.001^**^
	Active Experimentation (AE)	0 %	100 %	25.0 %	8.3 %	<0.001*
	Reflective Observation (RO)	0 %	100 %	35.7 %	28.6 %	<0.001*
	Concrete Experience (CE)	0 %	100 %	15.8 %	15.0 %	<0.001*
	**Total**	0 %	100 %	23.8 %	19.0 %	<0.001*

* Highly significant (p < 0.01).

All cohorts attained 100 % AED competence immediately post-training (Table 4**)**. Over time, only the Visual subgroup and both framework totals changed significantly (p < 0.001), whereas Auditory and Kinesthetic shifts were non-significant (p = 0.157/0.172). At 6 months Kolb learners out-performed VAK (66.7 % vs 34.8 %), driven by significant gains in AC, AE, RO and CE (p ≤ 0.018). By 9 months performance improved overall—VAK 63.6 %, Kolb 71.4 %—with Reflective Observation leading (85.7 %) and Abstract Conceptualization lowest (61.1 %), both still highly significant.

**Table 4:** Total level of satisfactory AED performance by learning style.

**Model**	**Learning Style**	**Pre Test**	**Immediate Post Test**	**6 Mo Post** **Test**	**9 Mo Post Test**	***p*–value**
		**VAK n = 67,Kolb n = 67**	**VAK n = 67,Kolb n = 67**	**VAK n = 66,Kolb n = 63**	**VAK n = 66,Kolb n = 63**	
**VAK**	Visual	23.2 %	100 %	27.3 %	60.0 %	<0.001*
	Auditory	20.0 %	100 %	66.7 %	83.3 %	0.157
	Kinesthetic	0 %	100 %	80.0 %	80.0 %	0.172
	**Total (VAK)**	20.9 %	100 %	34.8 %	63.6 %	<0.001*

**Kolb**	Abstract Conceptualization (AC)	21.1 %	100 %	61.1 %	61.1 %	0.002*
	Active Experimentation (AE)	15.4 %	100 %	66.7 %	75.0 %	0.018*
	Reflective Observation (RO)	14.3 %	100 %	78.6 %	85.7 %	0.007*
	Concrete Experience (CE)	42.9 %	100 %	63.2 %	68.4 %	0.001*
	**Total (Kolb)**	25.4 %	100 %	66.7 %	71.4 %	<0.001*

* Highly significant (p < 0.01).

Regression results (Table 5**)** indicate that, because VAK- and Kolb-classified participants received the same multimodal CPR program, the VAK vs Kolb coefficient merely reflects learner-group coding; coefficients were small across outcomes (|β| ≤ 0.26). Age was positively associated with knowledge (β = 0.54) and AED skills (β = 0.50) but inversely with BLS skills (β = −0.58). Male sex linked to higher BLS scores (β = 1.06) and minimal effects elsewhere. Higher education showed a modest negative association with knowledge (β = −0.63) and little impact on skills. Prior real-life CPR participation related to higher knowledge (β = 0.39) but lower BLS scores (β = −1.56). Years of experience had negligible associations.

**Table 5:** **Multiple-linear regression: predictors of 9-month CPR outcomes.** (unstandardized β-coefficients; Kolb = reference group).

**Outcome**	**β (VAK vs Kolb)**	**β (Age)**^a^	**β (Sex)**^b^	**β (Education)**^c^	**β (Real-life CPR)**^d^	**β (Experience)**^e^
Knowledge (0–15)	0.23	0.54	–0.14	–0.63	0.39	–0.05
BLS skills (0–30)	0.26	–0.58	1.06	–0.04	–1.56	0.12
AED skills (0–10)	–0.19	0.50	–0.37	0.23	–0.03	–0.06

Model adjusts simultaneously for all covariates (n = 129 complete cases); intercepts omitted for brevity.

a Age coded ordinally: 25–34 yr = 1; 35–44 yr = 2; 45–54 yr = 3; 55–64 yr = 4.

b Sex: Male = 1, Female = 0.

c Education level: Bachelor of Science in Nursing = 1, Diploma/Associate = 0.

d Previous participation in a real-life arrest with CPR performed: yes = 1, No = 0.

e Years of experience coded as band mid-points: <1 yr = 0.5; 1–5 yr = 3; 6–10 yr = 8; 11–15 yr = 13; >15 yr = 17.

**Fig. 2:**
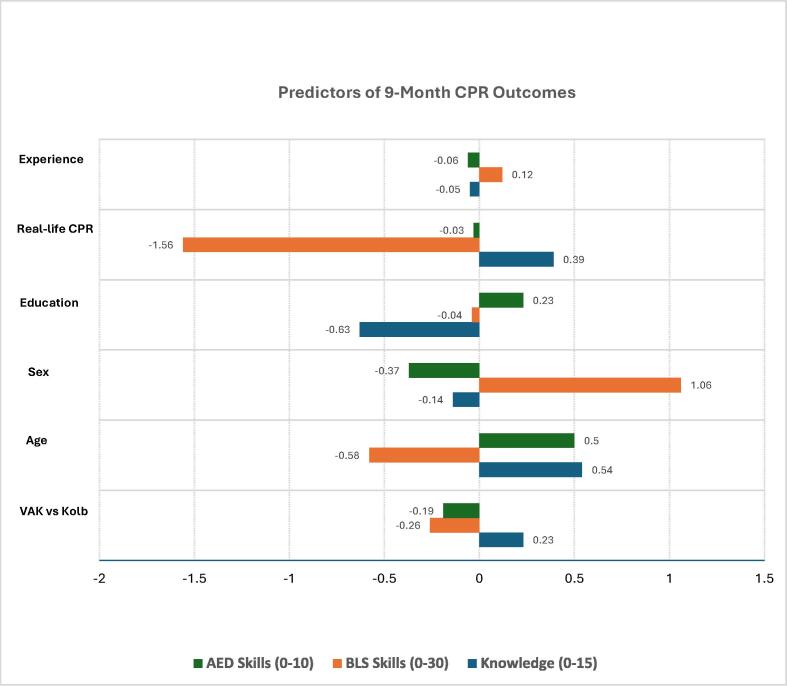
Predictors of 9-Month CPR Outcomes.

As illustrated in Fig. 2, VAK learners scored modestly higher than Kolb learners on 9-month knowledge (+0.23) and BLS totals (+0.26) but slightly lower on AED tasks (−0.19). Each step up in age band added about half a knowledge or AED point (+0.54, +0.50) yet corresponded to a small BLS loss (−0.58). Male sex showed the largest single effect—a +1.06 gain on the 30-item BLS checklist—while nudging knowledge and AED scores downward (−0.14, −0.37). BSN education linked to fewer correct knowledge items (−0.63) and negligible change in hands-on metrics. Real-life arrest experience improved knowledge (+0.39) and AED (+0.23) but aligned with a −1.56 drop in demonstrated BLS steps, whereas overall years of practice contributed little (β ≈ 0).

## Discussion

The cohort was predominantly female with more than three quarters under 45 years and had lower bachelor-level attainment than U.S. and international benchmarks^17^, ^18^, ^19^. Similar demographics are reported in Canada, the U.K., and across the Arab Gulf.^20^ Most nurses had <10 years’ experience, consistent with regional data from Al Boliteeh et al.^21^. Despite limited tenure, less than two thirds had already managed a cardiac arrest, paralleling findings by Danielsen et al.^22^ and Hotan & Meshary.^23^

In relation to VAK and KOLB Learning styles distribution within each group. The majority of them were Visual learners, contrasting with the kinesthetic-led pattern in the Egyptian survey by Ebrahim & Thabet^24^ and the Saudi study by Mariano et al.^25^ Kolb preferences were more evenly distributed, with a slight Concrete Experience (CE) lead; this diverges from the Diverger-heavy student sample described by Campos et al.^26^

Concerning CPR Knowledge retention, baseline BLS knowledge was fragmented: the vast majority of them recalled the 30: 2 ratios, yet only less than one third understood CPR purpose—echoing the Egyptian audit by Zayed & Saied^27^ and the Bahraini survey by Alsabri et al.^28^ Scores plummeted at six months (≈36–48 % correct), mirroring the decay reported by Shokry Abd Allah et al. in Egypt^29^ and Mohideen & Ablao in Saudi Arabia.^30^ A modest rebound at nine months—most pronounced in scenario-based items—parallels observations by Marzooq et al.^31^ and Elhak et al.^32^

Regarding VAK effects, at baseline no auditory or kinesthetic learners met the ≥60 % benchmark; after training, all did. Six months later, kinesthetic learners still scored 100 %, versus 22 % visual and 0 % auditory, confirming that hands-on strategies best preserve psychomotor memory (see Praise et al.,^33^ Haynes et al.,^34^ Nicolau et al.,^35^ and motor learning work by Wulf & Lewthwaite.^36^ Although the χ^2^ test was not statistically significant, the 73–point decline we observed echoes the sharp post–training fade—and the recovery that follows timely refreshers—described in Wulf & Lewthwaite’s OPTIMAL–theory experiments^37^; the Omani six–month booster trial by Jadidi & Jufaili^38^; nursing–student cohorts in *Resuscitation Plus* using the RQI® curriculum (Oermann et al.)^39^; the NIRS study of cerebral autoregulation after cardiac arrest by Ameloot et al.^40^; the Jordanian simulation RCT by Abu–Wardeh et al.^41^; the Saudi debriefing trial by Gaafar et al.^42^; the year–long Turkish follow–up by Bukiran et al.^43^; the Canadian airway–skills maintenance trial by Kovacs et al.^44^; and the German learning–style cohort reported by Schroeder et al.^45^

Regarding VAK effects, at baseline no auditory or kinesthetic learners met the ≥60 % benchmark; after training, all did. Six months later, kinesthetic learners still scored 100 %, versus 22 % visual and 0 % auditory, confirming that hands-on strategies best preserve psychomotor memory (see Praise et al.,^33^ Haynes et al.,^34^ Nicolau et al.,^35^ and motor-learning work by Wulf & Lewthwaite.^36^ Although χ^2^ was non-significant, the 73-point drop mirrors the Omani trial by Jadidi & Jufaili—where a six-month booster preserved competence.^38^, ^39^, ^40^, ^41^

Regarding Kolb effects, CE learners led at baseline (≥60 %), but post-training scores converged (86–95 %). Mark et al. (2025), in a Jordanian quasi-experimental study, likewise showed that style-aligned CPR training equalized performance immediately and sustained gains at three months.^41^ By nine months, Reflective Observers (RO) retained the most knowledge, whereas CE and Active Experimenters (AE) decayed faster, echoing the Egyptian findings of Zayed & Saied and the Gulf data of Jadidi & Jufaili.^27^, ^38^

Concerning skill trajectory, psychomotor scores rose from <50 % to >95 % after one four-hour session, as reported by Zayed & Saied^27^ and replicated by Praise et al.^33^ Scores halved by six months—matching the Saudi RCT by Gaafar et al.^42^—and scene-safety steps eroded further by nine months, whereas compression quality partly rebounded, potentially because several nurses reported performing real-life compressions or in-unit mock codes, which may have refreshed muscle memory even in the absence of formal retraining.^43^, ^44^, ^45^

Regarding AED performance, only 11 % could power on an AED at baseline—similar to observations in U.K. care homes by Plant & Taylor and Malaysian clinics by Htay et al.—yet post-training scores exceeded 94 %.^46^ Six-month retention fell to ≈ 28 %, paralleling the Saudi survey by Al Radini et al.^47^ Both the Omani booster trial of Jadidi & Jufaili and the Egyptian simulation study by Mostafa et al. show that feedback-driven micro-refreshers maintain ≥ 90 % AED competence at 12 months.^38^, ^46^

Collectively, across VAK and Kolb profiles, CPR and AED competence can decline by up to 80 % within nine months without reinforcement. Multicenter evidence—from Mark et al., Jadidi & Jufaili, Mostafa et al., and others in Qatar, Egypt, Oman, and Saudi Arabia—consistently calls for quarterly, feedback-rich drills to sustain bedside-ready performance.^38^, ^43^, ^44^, ^45^, ^46^, ^47^, ^48^, ^49^, ^50^ Echoing the rapid post–training decay described by Ali et al. and Abd El Naeem et al. who found that nurses’ CPR competence can fall back toward baseline within as little as six months, Our findings indicate that tailoring refresher intervals to each nurse’s dominant learning modality sustains high–quality performance for at least nine months, thereby preventing the usual post–training drop–off.^51^, ^52^

Proponents of learning-style matching contend that aligning instructional modalities with individual preferences—such as tailoring visuals for visual learners or hands-on drills for kinesthetic learners—can accelerate initial skill acquisition and improve learner satisfaction, a position supported by empirical work on VAK applications and academic performance outcomes in health-care education,^11^, ^12^ however, broader scoping reviews reveal limited evidence that strict matching yields durable performance gains and caution that over-reliance on preferred modalities may reduce cognitive flexibility.^13^ Motor-learning and simulation studies suggest an alternative “desirable difficulty” approach, whereby deliberate exposure to non-preferred formats (e.g., auditory briefs for visual learners) fosters deeper encoding and long-term retention through increased attentional demand and intrinsic motivation.^37^ Low-dose, high-frequency simulation trials further demonstrate that varied, feedback-rich practice sustains CPR competence more effectively than single-modality refresher courses.^34^, ^35^ Collectively, this evidence supports an adaptive blend: begin with style-aligned content to engage learners, then rotate modalities in subsequent refreshers to build resilience and promote cross-modal transfer, thereby reconciling the advantages of both matching and strategic deviation.

Our findings carry life-saving significance: because survival after out-of-hospital cardiac arrest falls by roughly 7–10 % for every minute that CPR or defibrillation is delayed,^53^ closing the competence gaps we uncovered—and implementing the targeted interventions we outline—can directly preserve lives. In Qatar, Emergency Medical Services generally arrive 5–10 min after the call,^54^ any break in nurse-initiated CPR during this window can therefore halve a patient’s chances. Our data show that visual, auditory and concrete-experience nurses lose up to 80–90 % of their skills within six months, whereas kinesthetic and reflective learners retain the most. Quarterly, feedback-rich refreshers are thus warranted: multicenter trials, including the Omani six-month booster study, consistently restore ≥90 % competence and require less time away from duty than annual courses.^38^ Focusing these sessions on the fastest-declining subgroups is cost-neutral yet yields the greatest clinical benefit. Finally, sustained, documented competence aligns with Joint Commission resuscitation standards mandating ongoing training and mock-code participation for accredited organizations,^55^ appointing a dedicated resuscitation officer within Private Nursing Services in Qatar would operationalize this requirement and reduce organizational risk.

## Conclusion

Retention trajectories varied markedly by learning style. Visual, auditory, and concrete-experience learners attained near-universal mastery immediately after training but experienced the quickest skill erosion, dropping to moderate competence within six months. By nine months, VAK-kinesthetic learners and Kolb reflective observers still held the strongest CPR and AED performance, whereas VAK-visual, VAK-active, and Kolb abstract-conceptualizer groups showed the steepest declines. Thus, long-term competence depends not only on refresher intervals but also on the cognitive-sensory channels through which nurses originally learn.

### Recommendations

Incorporating formal VAK and Kolb profiling into the initial competency assessment of home-care nurses would allow refresher schedules to be tailored to each practitioner’s dominant learning modality. Visual, auditory, and concrete-experience learners whose competence fell below the satisfactory threshold within six months, they should engage in quarterly, low-dose, high-frequency refreshers, whereas kinesthetic and reflective learners, who retained skills for longer, could engage in semi-annual refreshers; the full team should still undergo a comprehensive annual audit. Establishing a dedicated Resuscitation Officer service to the private home-care sector in Qatar would ensure consistent coordination of these schedules, rigorous performance monitoring, and standardized practice. A formal six-month skills audit using objective tools should guide clinical deployment, and refresher engagement should be linked to real patient outcomes. Lastly, further research is needed to validate these strategies across diverse settings and assess their cost-effectiveness and impact on survival.

### Limitations

This study has limitations that warrant acknowledgment but do not compromise the overall integrity of its findings. The focus on a home-care nurse cohort in Qatar limits generalizability to broader populations. Small subgroup sizes for auditory and active-experimenter learners reduced the power for detailed comparisons. Self-reported learning styles may introduce bias, and skill assessments conducted in simulations could overestimate real-world competence. Additionally, outcomes were educational in nature and not linked to clinical endpoints such as return of spontaneous circulation (ROSC) or survival.

## Provenance and peer review

Not commissioned; externally peer–reviewed.

## Consent for publication

All authors have read and approved the final version of the manuscript and consent to its publication in *Resuscitation Plus*. No individual person’s data (images, videos, or personal details) are presented that would require separate consent.

## Source of funding

Self-Funding.

## Data Availability

Data are freely available in the paper and its supporting information. De–identified study data, study protocol, and analysis code are openly available and can be obtained from the corresponding author upon reasonable request.

## CRediT authorship contribution statement

**Mohamed Elsayed Saad aboudonya:** Writing – original draft, Visualization, Validation, Investigation, Formal analysis, Data curation, Conceptualization. **Hoda Diab Fahmy Ibrahim:** Writing – review & editing, Validation, Supervision, Methodology, Data curation, Conceptualization. **Safaa R. Osman:** Writing – review & editing, Validation, Supervision, Project administration, Methodology, Formal analysis, Data curation, Conceptualization.

## Declaration of competing interest

The authors declare that they have no known competing financial interests or personal relationships that could have appeared to influence the work reported in this paper.
