# Corrigendum to “Influence of an educational program utilizing VAK and Kolb’s learning theories on basic cardiopulmonary resuscitation knowledge and practices among private home nurses in Qatar” [Resuscitation Plus 26 (2025) 101071]

**DOI:** 10.1016/j.resplu.2025.101188

**Published:** 2025-12-17

**Authors:** Mohamed Elsayed Saad Aboudonya, Hoda Diab Fahmy Ibrahim, Safaa R. Osman

**Affiliations:** a Hamad International Training Center, Qatar; b Faculty of Nursing, Assiut University, Egypt

The authors regret, the support of the Medical Research Center at Hamad Medical Corporation, Doha, Qatar, for funding the publication of this study (Protocol MRC-01-24-266).

The authors would like to apologise for any inconvenience caused.

