# Supplementary material for: Influence of an educational program utilizing VAK and Kolb’s learning theories on basic cardiopulmonary resuscitation knowledge and practices among private home nurses in Qatar
Source: Resusc Plus. 2025 Aug 19;26:101071. doi: 10.1016/j.resplu.2025.101071 (PMC12421586; doi:10.1016/j.resplu.2025.101071)
Supplement: Supplementary Data 1 [file mmc1.docx]

**Supplementary Table S1**

**The table is explaining the Alignment of Training Components with VAK and Kolb Learning Styles' expanding how visual/auditory cues, hands‑on practice, reflection, and experimentation were specifically embedded in the lecture and ERC four‑stage workshop.**

| Program components: | VAK Learning style(s) | Kolb learning-cycle stage(s) activated | Kolb learner types served | Why it resonates / expected benefit |
| --- | --- | --- | --- | --- |
| 1. Interactive PowerPoint + embedded full-scenario video (airway check, compressions, AED use) | - Visual (slides, video) - Auditory (narration, sound cues) | - Abstract Conceptualization (AC) – rules & algorithms - Reflective Observation (RO) – watching a complete scenario unfold | - Assimilators, - Divergers | Dual-coding (image + sound) builds a mental model; video realism triggers emotional memory and primes later hands-on practice. |
| 1. Facilitated discussion of real-life reflective scenarios (What went well? What failed? How to improve?) | - Auditory (dialogue) - Visual (case images) | - RO – analyzing experience - AC – distilling principles | - Divergers, - Reflective Observers | Storytelling plus peer reflection deepens meaning and links classroom content to nurses’ lived experiences. |
| 1. ERC four-stage skills demonstration 1 real time demo 2 Demo + talk-through 3 Guided practice 4 Unassisted performance with feedback | - Stage 1–2: Visual + Auditory - Stage 3–4: Kinesthetic (hands-on) | - Concrete Experience (CE) – doing compressions/AED - Active Experimentation (AE) – adjusting technique on the spot | - Accommodators, - Convergers, - Kinesthetic learners | Physical rehearsal with immediate feedback encodes procedural memory; repetition across stages reinforces every sensory channel, explaining the strong retention seen in kinesthetic and active learners. |
